# Comment on ‘Comparative effectiveness of nonpharmacological interventions in reducing psychological symptoms among patients with chronic low back pain’

**DOI:** 10.1097/JS9.0000000000001491

**Published:** 2024-04-23

**Authors:** Yanwei You, Yuquan Chen, Alimjan Ablitip, Xindong Ma

**Affiliations:** a Division of Sports Science and Physical Education, Tsinghua University; b School of Social Sciences, Tsinghua University, Beijing, People’s Republic of China; c School of Public Health and Preventive Medicine, Faculty of Medicine, Nursing & Health Sciences, Monash University, Melbourne, Australia

*Dear Editor,*

**Figure 1:**
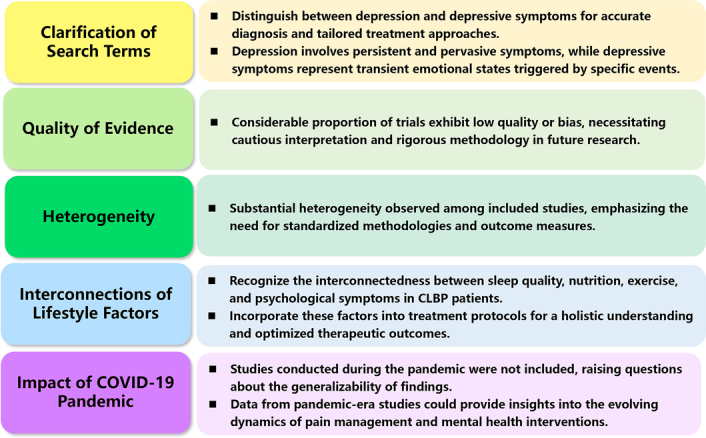
Major concerns in Zhou *et al*.’s study.

We are writing to commend the recent publication titled ‘Comparative effectiveness of nonpharmacological interventions in reducing psychological symptoms among patients with chronic low back pain’ in *International Journal of Surgery* ^1^. This systematic review and network meta-analysis shed light on an essential aspect of clinical management, addressing the psychological symptoms associated with chronic low back pain (CLBP). The findings of this study offer valuable insights into the management of CLBP, a condition that significantly impacts patients’ quality of life and often coexists with psychological symptoms. The identification of effective interventions for alleviating depression, anxiety, and improving mental health in CLBP patients has significant implications for clinical practice. However, several issues require further clarification, as concluded in Figure 1.

First, as for search term precision (Appendix 1), this study confused the concept of depression with ‘depressive symptom’ or ‘depressive mood’. It’s crucial to distinguish between depression and depressive symptoms, as they represent distinct psychological states with varying implications for clinical management. Depression, classified as an affective disorder, encompasses persistent and pervasive symptoms such as prolonged feelings of sadness, loss of interest or pleasure, helplessness, low self-esteem, diminished concentration, and disrupted social functioning^2^. On the other hand, depressive symptom represents a transient and less severe emotional state often triggered by specific events or circumstances, such as failure, disappointment, grief, or setbacks^3^. Therefore, when interpreting research findings and designing interventions, it’s essential to differentiate between depression and depressive symptoms to ensure accurate diagnosis and tailored treatment approaches.

Second, while the study provides valuable insights, it is essential to acknowledge the limitations associated with the quality of evidence. The quality of evidence was typically low or exhibited some risks of bias (47 out of 66 trials, 71.3%) in a considerable proportion of trials. This highlights the need for cautious interpretation of the results and underscores the importance of rigorous methodology in future research endeavors.

Third, as for heterogeneity: substantial heterogeneity observed among the included studies warrants careful consideration. For instance, Appendix 15 (depression), 20 (anxiety) and 25 (mental health) in this study showed high heterogeneity (*I* ^2^>50%). The variation in study designs, nonpharmacological interventions, and measures of psychological symptoms may contribute to the observed heterogeneity and could potentially impact the precision of summary estimates. Future studies should aim to minimize heterogeneity through standardized methodologies and outcome measures.

Fourth, it is imperative to recognize the interconnectedness between sleep quality, nutrition, exercise, and psychological symptoms in CLBP patients. While the study focuses on nonpharmacological interventions, a comprehensive approach should incorporate factors such as sleep hygiene, dietary patterns, and physical activity levels. Systematic consideration of these lifestyle factors can provide a holistic understanding of their impact on CLBP and associated psychological distress. Future research endeavors should prioritize large-scale, real-world studies to elucidate the complex interplay between these variables and expand upon the findings of the current study^4^. By integrating sleep, nutrition, and exercise into treatment protocols, clinicians can adopt a multifaceted approach to address the diverse needs of CLBP patients and optimize therapeutic outcomes.

Fifth, it is worth noting that studies conducted during the pandemic were not included in this analysis. This omission raises questions about the generalizability of the findings to the current healthcare landscape, where factors like remote interventions and altered healthcare delivery may have influenced the effectiveness of nonpharmacological interventions for chronic low back pain and associated psychological symptoms. Including data from studies conducted during the pandemic could provide valuable insights into the evolving dynamics of pain management and mental health interventions in the context of unprecedented healthcare challenges^5^.

Last but not least, I have some recommendations for clinical practice: While the study identifies several interventions such as mind–body therapy and a biopsychosocial approach that demonstrate effectiveness in reducing psychological symptoms among CLBP patients, it is crucial to consider the clinical applicability of these findings. Clinicians should weigh the potential benefits and risks of each intervention carefully, taking into account individual patient characteristics and preferences. Moreover, longitudinal studies assessing the long-term outcomes of these interventions would provide valuable insights into their sustainability and durability.
